# Evaporation and deposition of inclined colloidal droplets

**DOI:** 10.1038/s41598-021-97256-w

**Published:** 2021-09-07

**Authors:** Jin Young Kim, Marta Gonçalves, Narina Jung, Hyoungsoo Kim, Byung Mook Weon

**Affiliations:** 1 Research Center for Advanced Materials Technology, Sungkyunkwan University, Suwon, 16419 South Korea; 2 Department of Materials, ETH Zürich, 8093 Zurich, Switzerland; 3 SKKU Advanced Institute of Nanotechnology (SAINT), Sungkyunkwan University, Suwon, 16419 South Korea; 4 Department of Mechanical Engineering, Korea Advanced Institute of Science and Technology (KAIST), Daejeon, 34141 South Korea; 5 School of Advanced Materials Science and Engineering, Sungkyunkwan University, Suwon, 16419 South Korea

**Keywords:** Physics, Materials science, Soft materials

## Abstract

Colloidal droplets on flat solid substrates commonly leave symmetric ring-like deposits due to coffee-ring flows during evaporation. On inclined substrates, droplet shapes may become asymmetric by gravity. On this basis, it is not clear how their evaporation dynamics and final deposits are changed depending on inclination. Here we explore evaporation and deposition dynamics of colloidal droplets on inclined substrates, mainly by controlling colloidal particle size, substrate inclination, and relative humidity, which are crucial to gravitational intervention and evaporation dynamics. We experimentally investigate two different flows with opposite directions: downward sedimentation flows by gravity ($v_s$) and upward capillary flows by evaporation ($v_c$). We find that the competition of two flows determines the formation of final deposits with a flow speed ratio of $\alpha = v_s/v_c$. Notably, for $\alpha$ $\ll$ 1, evaporation-driven upward flows overwhelm sedimentation-driven downward flows, resulting in accentuated particle movement towards the top ring, which seems to defy gravitational intervention. We suggest a possible explanation for the flow speed dependence of final deposits in evaporating colloidal droplets. This study offers a framework to understand the intervention of inclination to the formation of final deposits and how to overcome the deposit pattern radial asymmetry, achieving symmetric deposit widths from inclined colloidal droplets.

## Introduction

Evaporation dynamics of colloidal droplets is an important process in many industrial and biological applications such as painting, inkjet printing, nanoparticle deposition, and biofabrication^1–7^. Understanding evaporation dynamics of colloidal droplets is essential to control and achieve desirable deposit patterns via evaporation-mediated processes^8–10^. Achieving desirable deposit patterns of colloidal droplets is not easy yet, because there are many complicated physical phenomena involved in evaporation and deposition dynamics of colloidal fluids^11–13^. For instance, coffee-ring effects are well-known phenomena by which colloidal particles tend to accumulate at triple-phase (solid, liquid, air) contact lines, leaving ring-like patterns during evaporation and thus disrupting formation of uniform deposit patterns^14–16^. Additionally, Marangoni flows^17–20^, wetting–dewetting transitions^21–24^, temperature variations^22,25,26^, and gravitational effects^26–31^ can modify the physical phenomena that govern evaporation and deposition dynamics of colloidal fluids.

External force fields, such as mechanical force or electromagnetic fields, can change the evaporation and deposition dynamics of colloidal droplets. Consider a general situation when a droplet is resting on a substrate; if the droplet radius is longer than a capillary length, it becomes distorted by gravity^32–36^. Then, depending on the gravitational field applied for the droplet, the colloidal particles inside the droplet can be significantly affected by gravity, and consequently, the final deposit patterns change during evaporation^27,37–40^. Any external force fields can somewhat modify the evaporation dynamics of colloidal droplets. However, understanding such external interventions is quite rare because of difficulty in direct observations of evaporating colloidal droplets under well-controlled external force fields, except for recent few studies^41,42^. Despite extensive studies on droplet evaporation dynamics, further studies are required to understand and control external interventions on evaporation and deposition dynamics of colloidal droplets.

In this article, we aim to identify the effect of gravity on evaporation and deposition dynamics of colloidal droplets by varying substrate inclination, colloidal particle size, and relative humidity. We explore the contribution of two main flows in evaporating droplets on inclined substrates: the sedimentation flow due to gravitational sedimentation of particles and the capillary flow by droplet evaporation. To figure out the competition of the two distinct flows, we experimentally control the inclination angles, the particle sizes, and the relative humidity, which modify the evaporation rates and thus the final deposit patterns. We clearly show how the final deposit patterns of colloidal droplets can be changed depending on the competition of the two (sedimentation and capillary) flows. If the capillary flow overwhelms the sedimentation flow, the particle deposition is more accentuated at the upper contact line than the lower contact line, which seems to defy the gravitational intervention. We propose a physical mechanism that accounts for the gravitation intervention to the conventional coffee-ring flows of evaporating droplets on inclined substrates. This result would be helpful to predict and manipulate the evaporation and deposition dynamics of colloidal droplets under the intervention of external forces, controlling the symmetry of the final pattern.

## Methods

In experiments, we used model colloidal suspensions to prevent dewetting during evaporation, including 2.5 wt% polystyrene non-functionalized colloidal particles with particle sizes ranging from 50 nm to 4.5 $\upmu$m (Polyscience Inc.). Same flat solid substrates consisting of $24 \times 50$ $\hbox {mm}^2$ cover glasses (Deckgläser) were used for all experiments. All the substrates were cleaned for 10 minutes in ethyl alcohol ($\ge$99.5% purity, No. 459844, Sigma-Aldrich) by using an ultrasonic cleaner (UC-10, Lab Companion) and dried air (99.99–99.999% purity) from a blowgun. For environmental conditions, the temperature was controlled to be $20 \pm 1^{\circ }$C by a temperature chamber (TC30, Krüss), and relative humidity was controlled from 10% to 90% by a humidity chamber (HC10, Krüss). For real-time visualizations, the side-view images of sessile droplets were taken by the drop shape analyzer (DSA25, Krüss), where the temperature and humidity chamber are attached to the sample stage. The conditions inside the chamber were stabilized at least 1 hour prior to the experiments to minimize the air flow influence on droplet evaporation. From the side-view images, we measured the contact angles and radii of evaporating droplets using the texture analysis method^31^. To assess the dynamics of an inclined colloidal droplet, three inclination angles were tested $\phi = 30^{\circ}$, $60^{\circ}$, and $90^{\circ }$. The micropipette was used to gently put the individual colloidal droplets (3 $\upmu$l) on the substrates. For a 3 $\upmu$l colloidal droplet, the Bond number, $Bo = \frac{R^2\rho g}{\sigma }$ (where *R* is the initial contact radius, $\rho$ is the colloidal drop density, *g* is the gravitational acceleration, and $\sigma$ is the surface tension), is 0.4. This *Bo* value and the contact radius being larger than the capillary length indicate the susceptibility of a 3 $\upmu$l droplet to the gravitational effect when inclined^38^. The final deposit patterns were imaged with the upright optical microscopy (VH-Z100R, Keyence) and further analyzed with ImageJ software (Maryland, USA).

**Figure 1:**
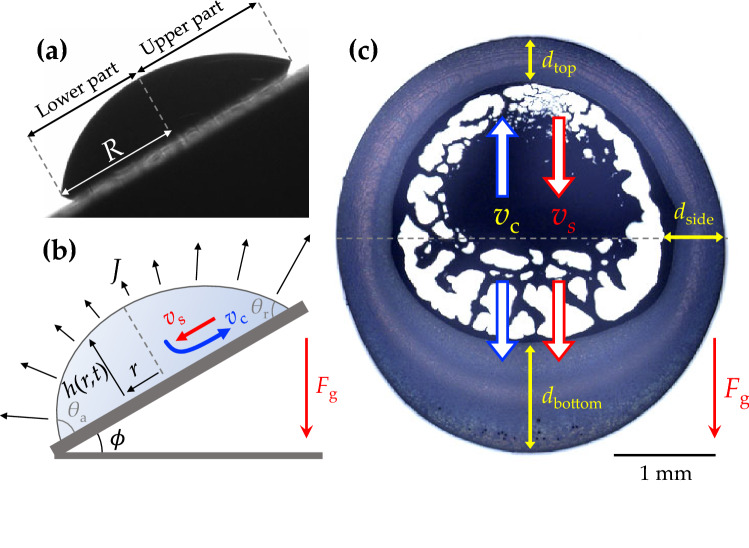
Evaporation and deposition of colloidal droplets on inclined substrates. (**a**) The side-view image of a 3 $\upmu$l colloidal droplet at the inclination angle $30^{\circ }$ taken with the drop shape analyzer. (**b**) A schematic illustration of a colloidal droplet at the inclination angle $\phi$ under gravity $F_g$. The droplet height *h*(*r*, *t*) depends on the position *r* and time *t*. The asymmetric droplet geometry affects the local evaporation flux *J*: typically, the evaporation flux is greater at the small contact angle ($\theta _r$) than at the large contact angle ($\theta _a$). For each position, the arrows mark the difference in evaporation flux. The two flows, $v_s$ and $v_c$, represent the sedimentation flow and the capillary flow, respectively. (**c**) The final deposit from (**a**) after evaporation. The two (sedimentation and capillary) flows would contribute to the ring widths at the top ($d_{top}$), the bottom ($d_{bottom}$), and the side ($d_{side}$) positions. The two flows compete in the upper part and cooperate in the lower part in the evaporating droplet, as illustrated by the blue and red arrows.

**Figure 2:**
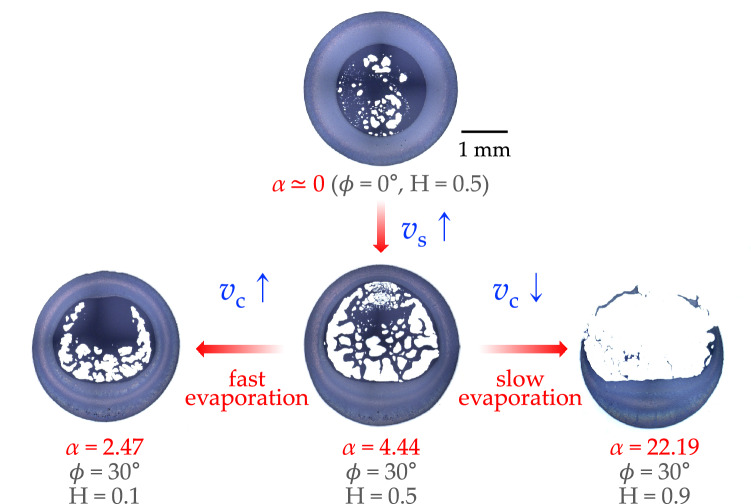
Final deposits of colloidal particles (particle diameter = 2 $\upmu$m) according to the relative humidity and the inclination angle. The speed ratio $\alpha$ is estimated from Eq.  (7).

## Results and discussion

### Sedimentation and evaporation in inclined colloidal droplets

We experimentally observed the final deposit patterns during and after evaporation of the colloidal droplets on the inclined substrates, as shown in Fig. 1. During evaporation, the colloidal particles move to follow the flows generated in the evaporating droplet, which causes the formation of the specific final pattern after evaporation. When the colloidal droplet evaporates on the non-inclined ($\phi = 0$) substrate, the final deposit pattern is determined mainly by the capillary flow that commonly leaves the ring-like deposits^14^. When the gravitation force $F_g$ is applied to the evaporation process by tilting the substrate (Fig. 1a), the two flows are generated as the gravity-driven downward sedimentation flow (with speed $v_s$) as well as the evaporation-driven edgeward capillary flow (with speed $v_c$) (Fig. 1b). The direction of the sedimentation flow is identical to the direction of gravity, while the capillary flow radially moves toward the contact line. Consequently, the two flows would result in the ring widths at the top ($d_{top}$), the bottom ($d_{bottom}$), and the side ($d_{side}$) positions. The two flows compete in the upper part and cooperate in the lower part in the evaporating droplet (Fig. 1c). Inevitably, the top ring becomes narrow by the competition of the two flows, and the bottom ring becomes wide by the cooperation of the two flows. This result implies that the gravitational intervention significantly contributes to the evaporation dynamics and the final deposit patterns.

To assess the gravitational intervention, we investigate the two different flows. From Stokes’ law, we obtain the terminal velocity of particle sedimentation in the droplet as given by^30^

1 $$\begin{aligned} v_s = \frac{(\rho _p - \rho _f)g D^2}{18 \mu } \sin \phi , \end{aligned}$$

where $\rho _p$ and $\rho _f$ are the densities of colloidal particles and fluids, respectively, *g* is the gravitational acceleration, *D* is the particle diameter, $\mu$ is the solvent dynamic viscosity, and $\phi$ is the substrate inclination angle.

To estimate the velocity of the capillary flow $v_c$, we apply the cylindrical coordinates (*r*, *h*) for the position *r* and the height of the droplet *h*, with assumption that the contact line of the droplet initially gets pinned by the self-pinning of highly concentrated colloidal particles (2.5 wt%)^16^. From the lubrication approximation, we adopt the continuity equation based on the mass conservation law in the evaporating droplet as^43^

2 $$\begin{aligned} \frac{\partial h(r,t)}{\partial t} = -\frac{1}{r}\frac{\partial Q}{ \partial r} - \frac{J(r)}{\rho _f}, \end{aligned}$$

where *h*(*r*, *t*) is the height of the droplet, expressed as $h(r,t) = \frac{R^2-r^2}{2R} \theta (t)$ from the geometric assumption of the parabolic-shaped droplet, *Q* is the volumetric flow rate, and *J*(*r*) is the local evaporation flux. For the diffusion-limited evaporation mechanism of the sessile droplet, *J*(*r*) can be expressed as^10^

3 $$\begin{aligned} J(r) = \frac{2}{\pi } \frac{D_v C_s (1-H)}{\sqrt{R^2 - r^2}}, \end{aligned}$$

where $D_v$ is the diffusion coefficient of the water vapor in air ($D_v \approx 2 \times 10^{-5}$ m^2^/s at room temperature), $C_s$ is the saturated water vapor concentration ($C_s \approx 2 \times 10^{1}$ g/m^3^ at room temperature), *R* is the contact radius of the droplet, and *H* is the relative humidity. Substituting Eq. (3) into Eq. (2), we get the volumetric flow rate as

4 $$\begin{aligned} Q = \int \left[ \left( -\frac{rR^2-r^3}{2R} \right) \frac{\partial \theta }{\partial t}-\frac{2D_vC_s (1-H)}{\pi \rho _f}\frac{r}{\sqrt{R^2-r^2}} \right] dr \end{aligned}$$

and at $r \approx 0$, near the middle of the droplet on the inclined substrate, *Q* can be simplified as

5 $$\begin{aligned} Q = \frac{2RD_vC_s(1-H)}{\pi \rho _f}. \end{aligned}$$

From Eq. (5), we obtain the mean flow velocity at the cross-sectional area of the droplet as

6 $$\begin{aligned} v_c \simeq \frac{Q}{Rh} \simeq \frac{4D_vC_s(1-H)}{\pi \rho _f R} \frac{1}{\theta (t)}. \end{aligned}$$

To access the relative contribution of the two (sedimentation and capillary) flows, we suggest the flow speed ratio from Eqs. (1) and (6) as

7 $$\begin{aligned} \alpha = v_s/v_c = \omega \cdot \frac{D^2\sin \phi }{(1-H)} \end{aligned}$$

where $\omega$ is a constant estimated as $\omega = \frac{\pi g(\rho _{p} - \rho _{f})\rho _{f} R \theta (t)}{72 \mu D_{v}C_{s}}$. We focus on the competition of the two flows at the upper part of the droplet. By comparing $\alpha$, we are able to expect which flow becomes dominant leading to preferential particle accumulation at the top and bottom contact lines. At $\alpha > 1$ (when the sedimentation flow is dominant), the colloidal particles are compacted to narrow the top ring; and vice versa, at $\alpha < 1$ (the capillary flow is dominant), the colloidal particles are accumulated to broaden the top ring. This explanation agrees with our experimental observations in Fig. 1c, where the sedimentation flow is dominant.

### Asymmetric deposit widths from inclined colloidal droplets

To understand the effect of the speed ratio $\alpha$ on the final deposit patterns, we investigate the phase diagram for the colloidal particles with the diameter of 2 $\upmu$m by varying the inclination angle $\phi$ and relative humidity *H* in Fig. 2. When the droplet evaporates on the non-inclined substrate at $\phi = 0^{\circ }$, the final deposit is radially symmetric because the coffee-ring effect is predominant against the sedimentation effect ($\alpha = 0$). However, at $\phi = 30^{\circ }$ (when the sedimentation effect becomes strong), the final deposit becomes asymmetric, and particularly the bottom ring becomes broader than the top ring of the droplet because of the sedimentation effect enhancement ($\alpha > 0$). In detail, as the relative humidity (*H*) changes from 0.9 to 0.1 at $\phi = 30^{\circ }$, the relative contribution of the evaporation-driven capillary flow becomes strong ($\alpha$ changes from 22.19 to 2.47). At *H* = 0.1, the droplet evaporates rapidly, and the relative effect of $v_c$ becomes stronger ($\alpha = 2.47$) so that the particles preferentially move upward towards the top contact line. At *H* = 0.9, the droplet evaporates slowly, and the relative effect of $v_c$ becomes weak ($\alpha = 22.19$) so that most sediments appear at the bottom ring by the sedimentation effect enhancement.

**Figure 3:**
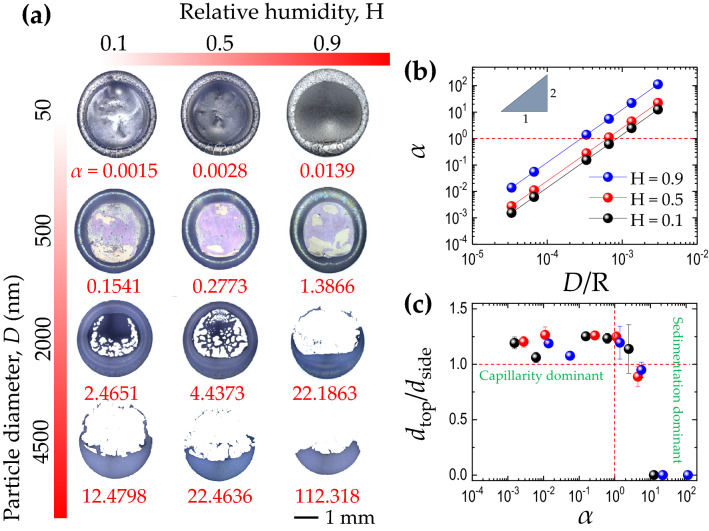
Phase diagram of final deposits at the inclination angle $\phi = 30^{\circ }$ according to the relative humidity and the particle diameter. (**a**) The final deposit patterns depend on the speed ratio $\alpha$, taken from the two conflicting (sedimentation and capillary) flows. (**b**) The power-law scaling of $\alpha$ with the particle diameter (*D*) divided by the droplet radius (*R*). (**c**) The top/side ring width dependence on $\alpha$. At $\alpha \gg 1$, the sedimentation effect is much dominant and the top ring is negligible.

**Figure 4:**
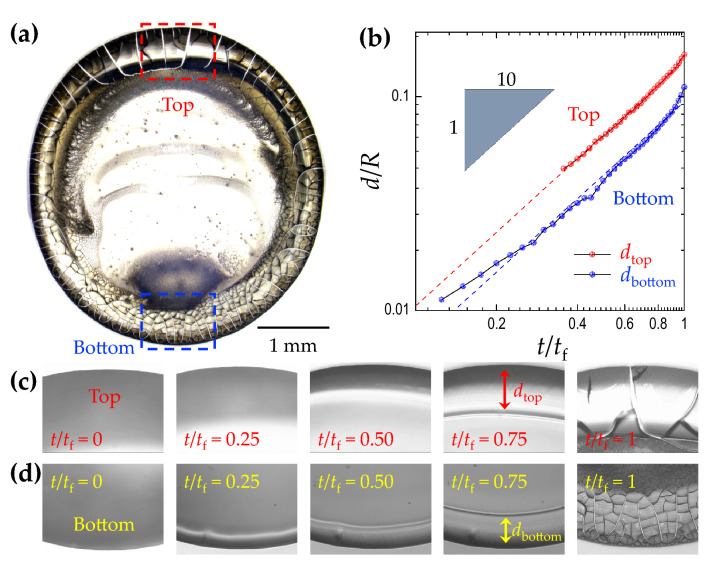
Ring width growth during evaporation. (**a**) The top-view image of the final deposit for $\phi = 90^{\circ}$, $D = 50$ nm, and $H = 0.5$ after evaporation. (**b**) The top (bottom) ring width growth, $d_{top}$ ($d_{bottom}$) normalized by the droplet contact radius *R*, with the evaporation time, $t/t_f$ ($t_f$ is the complete evaporation time). (**c**) and (**d**) The up-close sequential images at the top and the bottom rings, respectively.

The entire phase diagram of the final deposits at the fixed $\phi = 30^{\circ }$ by varying the relative humidity and the particle diameter is summarized in Fig. 3a. As seen here, we find that the final deposit patterns strongly depend on the speed ratio $\alpha$, quantified from sedimentation and capillary flows. For the particle diameter (from 50 nm to 4.5 $\upmu$m) and the relative humidity (from 0.1 to 0.9) at the fixed $\phi = 30^{\circ }$, the bottom ring width increases as $\alpha$ increases. Particularly, at $\alpha > 1$, the final deposit becomes asymmetric by the enhanced sedimentation.

Importantly, we find the power-law scaling of $\alpha$ with the particle diameter divided by the droplet radius in Fig. 3b, regardless of the relative humidity. The power-law scaling of $\alpha$ is easily predicted from the definition of $\alpha = v_s/v_c$ in Eq. (7), suggesting the power-law scaling of $\alpha \propto D^2$, indicating that increment of particle size leads to stronger sedimentation flow. Intuitively, we know that the larger the particle size and the higher the relative humidity, the more significant the sedimentation effect thanks to the larger $\alpha$. Specifically, $\alpha = 1$ can be possible at $D = 1$ $\upmu$m, $\phi = 30^{\circ }$, and $H = 0.75$, indicating that both (sedimentation and capillary) flows become identical. Therefore, the critical condition for $\alpha = 1$ is predictable according to the particle diameter and the relative humidity.

The final ring width strongly depends on the $\alpha$ quantity, as demonstrated in Fig. 3c. At $\alpha > 1$, the sedimentation effect is dominant, leading to a broader bottom ring width. We measure the ratio of top and side ring widths, i.e., $d_{top}/d_{side}$, from the entire cases and find that the ring width ratio critically decreases to be $d_{top}/d_{side} \approx 0$ at $\alpha \gg 1$. In contrast, when $\alpha <1$, $d_{top}/d_{side}$ is always greater than 1. Consequently, the top ring width is strongly dependent on the gravitational sedimentation effect.

### Symmetric deposit widths from inclined colloidal droplets

The final deposit is a consequence of the local ring growth dynamics, as shown in Fig. 4a. From the real-time visualizations of the evaporating colloidal droplet, we directly measure the ring width growth of the top and the bottom rings and find that the top ring accumulates faster than the bottom ring (Fig. 4b). In this case, the particle size is very small and $\alpha =0.0055$, indicating that the sedimentation effect is negligible. The evaporation-driven capillary flow is then the dominant flow, leading to the preferential particle movement upwards. The up-close sequential images at the top and the bottom rings in Fig. 4c, d confirm that the top ring becomes wider than the bottom ring during evaporation. To check the accumulation dynamics of the particles at the top and the bottom ring, we normalize the ring width and the evaporation time with the droplet contact radius *R* and complete evaporation time *t*_*f*_, respectively, in the log-log plot, and the result shows the approximately power-law scaling (Fig. 4b). This observation is consistent with the previous numerical study^37^, suggesting $R \propto t^{\zeta /3}$ (*R* is the contact radius of the droplet and $\zeta = \frac{\pi -\theta (t)}{\frac{3}{4}\pi -\theta (t)}$). The slopes of the dashed lines in Fig. 4b are the theoretically obtained $\zeta$ values, being in good agreement with the experimentally obtained data.

**Figure 5:**
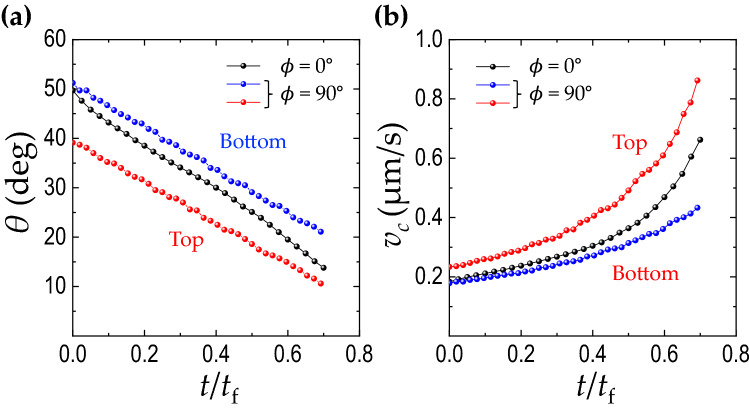
Contact angle influence on capillary flow velocity. (**a**) The temporal evolution of the measured contact angle for $\phi =0^{\circ }$ and $90^{\circ }$. (**b**) Theoretically obtained capillary flow velocity for $\phi =0^{\circ }$ and $90^{\circ }$. The black circles come from the non-inclined droplet, and red and blue circles are from the top and bottom contact point of the $90^{\circ }$ inclined droplet, respectively. This result suggests that the contact angle is crucial to determine the evaporation rate, leading to a wider deposit ring on the top section due to a dominant capillary flow.

**Figure 6:**
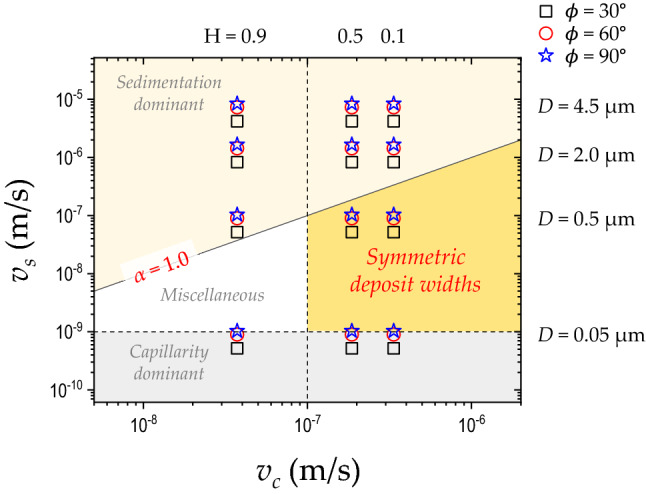
Phase diagram for symmetric deposit widths from inclined colloidal droplets. The symmetric deposit widths (bright yellow region), observed in total 27 data, are achieved at the low relative humidity ($H \lesssim 0.5$), with a particle diameter of 0.05 $< D < 2.0$ $\upmu$m, and the capillarity dominant regime ($\alpha < 1.0$), corresponding to $0.0055< \alpha < 1.0$. The asymmetric deposit widths are found in the other regimes (pale yellow and gray regions) and the mixed deposits in the miscellaneous regime (white region).

We consider the physical origin of the asymmetric deposit widths. At $\phi =0^{\circ }$ or $\phi =90^{\circ }$, the contact angles $\theta$ gradually decrease with time, as shown in Fig. 5a. We extract the capillary flow velocity in Eq. (6) from the real-time visualizations of the evaporating droplets. For $\phi =90^{\circ }$ inclination, the contact line is pinned and the contact radius is constant during evaporation, leading to contact angle hysteresis (that is, the contact angle on the top is quite smaller than on the bottom). As demonstrated in Fig. 5b, this contact angle asymmetry would inevitably drive the capillary flow asymmetry due to the enhanced evaporation rate at the top contact line. This result suggests that the final deposit pattern is indeed dependent on the dominance of the flows described here, being consistent with the present experimental observations.

Finally, we obtain the phase diagram for the symmetric deposit widths from the inclined colloidal droplets in Fig. 6. The symmetric deposit widths (bright yellow region) are experimentally observed in total 27 data at the low relative humidity ($H \lesssim 0.5$), mostly for particles with hundreds of nanometers diameter (0.05 $< D<$ 2.0 $\upmu$m), and the capillarity dominant regime ($\alpha < 1.0$). The asymmetric deposit widths are found in the other regimes characterized by sedimentation dominance (pale yellow region), capillary dominance (gray region), and the symmetric and asymmetric deposit widths in the miscellaneous regime (white region). From our experiments in Fig. 3a, we estimate the constant $\omega = 1.1 \pm 0.09$ $\upmu$ $\hbox {m}^{-2}$ in Eq. (7), producing $\alpha \approx \frac{D^2\sin \phi }{2(1-H)}$ (where $D^2$ has a unit of $\upmu$ $\hbox {m}^{2}$ and $\omega$ has a unit of $\upmu$ $\hbox {m}^{-2}$, so that $\alpha$ is a unitless quantity). The regime of the symmetric deposit widths corresponds to $0.0055< \alpha < 1.0$ in the phase diagram. Our results give a broad insight into the symmetric pattern width formation from the inclined colloidal droplets by controlling the sedimentation flow (the particle size *D* and the inclination angle $\phi$) and the evaporation rate (the relative humidity *H*).

## Conclusion

In conclusion, we show the gravity-induced inclination effect on the evaporation and deposition dynamics of colloidal droplets. The real-time visualizations and the theoretical considerations suggest that the asymmetric deposit widths originate from the gravitational intervention. Additionally, we show that the colloidal particles can accumulate more in the upward contact line, opposite to the gravitational force field. The relative contributions of the two flow speeds ($v_s$ and $v_c$) are quantitatively testable and predictable with the flow speed ratio ($\alpha$). To achieve the symmetric deposit widths from the inclined colloidal droplets, the conditions for $0.0055< \alpha < 1.0$ are preferable. Our work offers a comprehensive understanding of the effects of humidity combining with particle size and inclination angle on deposit patterns, comparing with recent works on the effects of particle size, substrate wettability, and inclination angle on deposit patterns^44^ and the effects of evaporation, substrate inclination, and surface roughness^45^. This finding would be helpful to understand and control the evaporation and deposition dynamics of colloidal fluids under the asymmetric external force fields.
